# Non-Metallic Biomaterials for Tooth Repair and Replacement. By Pekka Vallittu, Woodhead Publishing, 2013; 406 Pages. Price £145.00/US$245.00/€175.00 ISBN 978-0-85709-244-1

**DOI:** 10.3390/jfb4010001

**Published:** 2013-01-14

**Authors:** Shu-Kun Lin

**Affiliations:** MDPI AG, Kandererstrasse 25, CH-4057 Basel, Switzerland; E-Mail: lin@mdpi.com

The following paragraphs are reproduced from the website of the publisher [1]:

1. Discusses the properties of enamel and dentin and their role in adhesive dental restoration;
2. Chapters also examine the wear properties of dental ceramics, glasses and bioactive glass ceramics for tooth repair and replacement;
3. Dental composites and antibacterial restorative materials are also considered;
4. Provides a concise overview of non-metallic biomaterials for dental clinicians, materials scientists and academic researchers alike.

As the demand for healthy, attractive teeth increases, the methods and materials employed in restorative dentistry have become progressively more advanced. Non-metallic biomaterials for tooth repair and replacement focuses on the use of biomaterials for a range of applications in tooth repair and, in particular, dental restoration.

Part one reviews the structure, modification and repair of dental tissues. The properties of enamel and dentin and their role in adhesive dental restoration are discussed, along with biomineralization and biomimicry of tooth enamel, and enamel matrix proteins (EMPs) for periodontal regeneration. Part two goes on to discuss the processing, bonding and wear properties of dental ceramics, glasses and sol-gel derived bioactive glass ceramics for tooth repair and replacement. Dental composites for tooth repair and replacement are then the focus of part three, including composite adhesive and antibacterial restorative materials for dental applications. The effects of particulate filler systems on the properties and performance of dental polymer composites are considered, along with composite based oral implants, fibre reinforced composites (FRCs) as dental materials and luting cements for dental applications.

With its distinguished editor and international team of expert contributors, Non-Metallic Biomaterials for Tooth Repair and Replacement provides a clear overview for all those involved in the development and application of these materials, including academic researchers, materials scientists and dental clinicians.

## Table of Contents

### Part 1 Structure, Modification and Repair of Dental Tissues

Structure and properties of enamel and dentin.

V.P. Thompson, New York University College of Dentistry, USA:

1. Introduction;
2. Enamel;
3. Dentinoenamel-junction (DEJ);
4. Dentin;
5. Conclusion;
6. References.

Biomineralization and biomimicry of tooth enamel.

V. Uskoković, University of California, USA:

1. Introduction;
2. The structure of enamel;
3. Amelogenesis at the molecular scale;
4. Key issues in biomineralization and biomimicry of tooth enamel;
5. Conclusion;
6. Acknowledgments;
7. References.

Enamel and dentin bonding for adhesive dental restorations.

J. Perdigão, University of Minnesota, USA; and A. Sezinando, University Rey Juan Carlos, Spain:

1. New trends in restorative dentistry;
2. Dental adhesion;
3. Bonding substrates;
4. Current bonding strategies;
5. Dental adhesion mechanisms;
6. *In vitro versus In vivo* studies;
7. Incompatibility between adhesives systems and restorative materials;
8. Conclusion;
9. References.

Enamel matrix proteins (EMP) for periodontal regeneration.

N. Donos, UCL-Eastman Dental Institute, UK; H.D. Amin, Imperial College, London, UK; and X. Dereka, University of Athens, Greece:

1. Introduction to principles of periodontal regeneration;
2. Periodontal ligament (PDL) stem/progenitor cells;
3. Secretion and composition of enamel matrix proteins (EMP);
4. Modulation of cell differentiation by EMP and enamel matrix derivatives (EMD) *in vitro*;
5. *In vivo* studies (for bone regeneration);
6. Treatment of periodontal osseous defects with enamel matrix derivatives;
7. Conclusion;
8. References.

### Part 2 Dental Ceramics and Glasses for Tooth Repair and Replacement

Processing and bonding of dental ceramics.

J.P. Matinlinna, The University of Hong Kong, PR China:

1. Introduction to dental ceramics;
2. Alumina and zirconia chemistry;
3. Silane coupling agents and their chemistry;
4. Resin zirconia bonding;
5. Future trends;
6. Sources of further information and advice;
7. References.

Wear properties of dental ceramics.

M.K. Etman, University of Saskatchewan, Canada:

1. Introduction;
2. Clinical performance and wear of all-ceramic restorations;
3. *In-vitro* evaluation of wear and cracks in all-ceramic materials;
4. Conclusion;
5. References.

Sol-gel derived bioactive glass ceramics for dental applications.

X. Chatzistavrou, University of Michigan, USA; E. Kontonasaki, Aristotle University of Thessaloniki, Greece; K.M. Paraskevopoulos, Aristotle University of Thessaloniki, Greece; P. Koidis, Aristotle University of Thessaloniki, Greece; and A.R. Boccaccini, University of Erlangen-Nuremberg, Germany:

1. Introduction;
2. Sol-gel derived glasses and glass-ceramics;
3. Sol-gel derived coatings;
4. Sol-gel derived composites;
5. Conclusion and future trends;
6. References.

### Part 3 Dental Composites for Tooth Repair and Replacement

Composite adhesive restorative materials for dental applications. 

M.F. Burrow, The University of Hong Kong, PR China:

1. Introduction;
2. Resin composite restorative materials;
3. Polyacid-modified resin composite (compomer);
4. Glass ionomer (polyalkenoate) cements;
5. Resin modified glass ionomer cement (RM-GIC);
6. Conclusion;
7. References.

Antibacterial composite restorative materials for dental applications. 

I.M. Mehdawi, Benghazi University, Libya; A. Young, UCL Eastman Dental Institute, UK:

1. Introduction;
2. Current direct aesthetic restorative materials;
3. Antibacterial properties of aesthetic restorative materials;
4. Re-mineralizing dental composites;
5. Antibacterial, remineralizing and proteinases inhibiting materials;
6. Conclusion and future trends;
7. References.

Effects of particulate filler systems on the properties and performance of dental polymer composites. 

J.L. Ferracane, Oregon Health & Science University, USA; W.M. Palin, University of Birmingham, UK:

1. Introduction;
2. Current dental composite materials;
3. Theoretical considerations;
4. Types of fillers used in dental composites;
5. Effect of fillers on properties of dental composites;
6. Stability, degradation and clinical outcomes;
7. Current and future trends;
8. Sources of further information and advice;
9. References.

Composite based oral implants.

T.O. Närhi, University of Turku, Finland; A. Ballo, Gothenburg University, Sweden; and P.K. Vallittu, University of Turku, Finland:

1. Introduction;
2. Composition and structure;
3. Surface modification;
4. Biological response;
5. Clinical considerations and future trends;
6. References.

Fibre reinforced composites (FRCs) as dental materials.

P.K. Vallittu, University of Turku, Finland:

1. Introduction to fibre-reinforced composites (FRCs) as dental materials;
2. Structure and properties of FRCs;
3. Applications of FRCs in dentisty;
4. Fibre reinforced filling composites;
5. Future trends;
6. Conclusion;
7. References.

Luting cements for dental applications.

M. Özcan, University of Zurich, Switzerland:

1. Introduction;
2. Classification of cements;
3. Clinical implications of cement choice;
4. Conclusions and future trends;
5. References.

## * *Editor’s Note*:

The brief summary and the contents of the books are reported as provided by the authors or the publishers. Authors and publishers are encouraged to send review copies of their recent books of potential interest to readers of *Journal of Functional Biomaterials* to the Publisher (Dr. Shu-Kun Lin, Multidisciplinary Digital Publishing Institute (MDPI), Kandererstrasse 25, CH-4057 Basel, Switzerland. Tel. +41-61-683-77-34; Fax: +41-61-302-89-18; E-Mail: lin@mdpi.com). Some books will be offered to the scholarly community for the purpose of preparing full-length reviews.
